# Creating a Microenvironment to Give Wings to Dental Pulp Regeneration—Bioactive Scaffolds

**DOI:** 10.3390/pharmaceutics15010158

**Published:** 2023-01-03

**Authors:** Nan Hu, Weiping Li, Wentao Jiang, Jin Wen, Shensheng Gu

**Affiliations:** 1 Department of Endodontics, Shanghai Ninth People’s Hospital, Shanghai Jiao Tong University School of Medicine, College of Stomatology, Shanghai Jiao Tong University, Shanghai 200011, China; 2 National Center for Stomatology, National Clinical Research Center for Oral Diseases, Shanghai Key Laboratory of Stomatology, Shanghai Research Institute of Stomatology, Shanghai 200011, China; 3 Department of Oral and Maxillofacial Head & Neck Oncology, Shanghai Ninth People’s Hospital, College of Stomatology, Shanghai Jiao Tong University, Shanghai 200011, China; 4 Department of Prosthodontics, Shanghai Ninth People’s Hospital, Shanghai Jiao Tong University School of Medicine, College of Stomatology, Shanghai Jiao Tong University, Shanghai 200011, China; 5 Shanghai Key Laboratory of Stomatology, Shanghai Engineering Research Center of Advanced Dental Technology and Materials, Shanghai 200125, China

**Keywords:** dental pulp regeneration, bioactive scaffold, regeneration microenvironment

## Abstract

Dental pulp and periapical diseases make patients suffer from acute pain and economic loss. Although root canal therapies, as demonstrated through evidence-based medicine, can relieve symptoms and are commonly employed by dentists, it is still difficult to fully restore a dental pulp’s nutrition, sensory, and immune-regulation functions. In recent years, researchers have made significant progress in tissue engineering to regenerate dental pulp in a desired microenvironment. With breakthroughs in regenerative medicine and material science, bioactive scaffolds play a pivotal role in creating a suitable microenvironment for cell survival, proliferation, and differentiation, following dental restoration and regeneration. This article focuses on current challenges and novel perspectives about bioactive scaffolds in creating a microenvironment to promote dental pulp regeneration. We hope our readers will gain a deeper understanding and new inspiration of dental pulp regeneration through our summary.

## 1. Introduction

As a unique structure in our body, the tooth contains a hard shell and soft inner tissues to perform the chewing function. Once the hard shell is injured by caries or trauma, the outer bacterium will not only exert detrimental influences on the internal environment of the teeth but may also give rise to inflammation of the pulp or periapical tissue [1]. The onset of caries, pulpitis, and periapical periodontitis is more easily overlooked, so the incidence rate has been comparatively high in recent years. [2,3]. This results in painful experiences, tooth defects, and even tooth loss, which have long perplexed people tremendously. The conventional treatment method is root canal therapy, but that still leaves challenging problems such as persistent periapical infection, tooth discoloration, and heightened tooth brittleness [4,5,6]. Dental pulp regeneration with the participation of stem cells is an excellent way to resolve it. For some young permanent teeth, regenerative pulp therapy can partially be achieved by indirect or direct pulp capping, apexification, and revascularization [7]. However, the methods have limited effectiveness for mature permanent teeth, and further studies are still in the laboratory or clinical trial stage [8].

The tooth development process provides a favorable reference for pulp tissue engineering. In the seventh week of an embryo, the tooth lamina is formed in the primitive mouth. Then, mesenchymal stem cells from the ectodermal mesenchymal layer start to form dental papilla, which further develops into dentin and pulp through three continuing stages: the bud, cap, and bell phase [9]. This process is regulated by complex signals, such as TGF-β, Wnt, and FGF signaling pathways, during epithelial-mesenchymal interactions [10]. Furthermore, blood supply is vital for early tooth development. The papilla is infiltrated by multitudinous capillaries during the bell stage of embryonic development [1,11]. In a healthy and mature tooth, the vascular system predominantly contains supply arterioles, capillary networks, and venular networks, which constantly adapt to the nutritional, metabolic, and homeostatic needs of the tissues [1,12,13]. Regarding neurogenesis, pioneer nerve fibers ramify into the tooth germ and penetrate the inner dental papilla until dentinogenesis begins to form pulp tissue [9,14].

Teeth can regenerate once affected by caries, trauma, or other severe external stimuli. Odontoblasts around the dental pulp will secrete reparative dentin to cope with mild injuries. Nonetheless, suppose the damage is extensive and results in the death of odontoblasts; the mesenchymal stem cells will first redifferentiate into odontoblast-like cells and then secrete extracellular components to form restorative dentin subsequently [15]. This process is also regulated by many growth factors, such as TGF-β, IGF, and FGF2 [16,17,18]. When the damage continues to evolve, it will affect the dental pulp cells and the local blood supply and nerve function [19,20]. It is easy to obtain an oxygen-deficient environment since oxygen can travel about 100–200 microns through diffusion [21]. Such hypoxia conditions result in cells secreting pro-angiogenic factors that act on adjacent vascular endothelial cells to propel neovascularization. Unfortunately, this self-regulation is comparatively limited [22,23]. If the pulp continues to suffer from the insufficient blood supply, pulpitis and pulp necrosis may occur and even deteriorate to periapical periodontitis [24]. Meanwhile, peripheral nerve damage and gene expression changes will also occur, resulting in changes in pain thresholds and then give rise to various painful symptoms [25]. Consequently, it is paramount to maintaining a suitable microenvironment for dental pulp restoration and regeneration to enhance its regenerative capacity, whether the pulp is mildly irritated or even completely necrosis.

**Figure 1:**
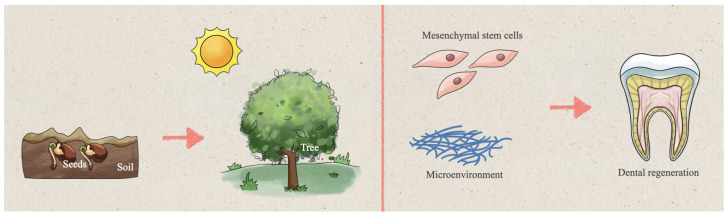
A schematic picture showing the relationship between mesenchymal stem cells and their microenvironment: the special microenvironment is to mesenchymal stem cells what soil is to seeds. Seeds grow slowly into trees under the nourishment of the soil just as mesenchymal stem cells strive to develop in their microenvironment, aiming to reach the goal of tissue regeneration.

The effects of tissue regeneration rest with mesenchymal stem cells and their microenvironment, just as plants’ growth depends on their healthy seeds and fertile soil (Figure 1) [26,27,28]. Dental stem cells are a large class of commonly used seed cells with the ability of cell proliferation and multi-differentiation, such as dental pulp stem cells (DPSCs), stem cells from the apical papilla (SCAPs), and stem cells from human exfoliated deciduous teeth (SHEDs), which have the potential to differentiating dental pulp [29,30,31,32]. The natural dental pulp microenvironment is crucial to maintaining the phenotype and differentiation potential of stem cells [33]. Specifically, the cellular microenvironment is the local and micro-scale environment of cell interaction, including soluble biochemicals, insoluble extracellular matrix, and surrounding cells, which is vital for regulating cell behavior and function [34,35,36]. It is well known that mesenchymal stem cells, scaffolds, and growth factors are the three critical components of tissue regeneration [37]. In other words, scaffolds and bioactive factors provide a favorable microenvironment and stimulate the differentiation of stem cells to propel pulp regeneration [38]. This paper primarily concentrates on forming bioactive materials with strategies from different angles to provide a favorable microenvironment for dental pulp regeneration.

## 2. Strategies

From January 2010 to October 2022, we conducted a literature search on the PubMed, ScienceDirect, and Web of Science databases, involving keywords such as “pulp regeneration”, “bioactive scaffold”, “regeneration microenvironment”, and “angiogenesis” to summarize strategies for creating a pulp regeneration microenvironment; 718 research articles were retrieved after removing duplicate records. Due to the wide range of retrieval and different methods for measuring the experimental research results, it is challenging to compare articles with each other to form a systematic literature review. Therefore, our review mainly includes 146 research articles on dental pulp regeneration microenvironmental scaffolds for a comprehensive narrative review, focusing on constructing bioactive scaffolds and their biological effects.

### 2.1. Natural and Naturally Derived Biomaterials

Many biological materials derived from natural tissues or cells have been reported, such as sodium alginate, gelatin, and collagen, which have been widely utilized in regenerative tissue engineering [39]. Here we will introduce some innovative, newly reported, tissue-specific bioactive materials that achieve satisfactory results through simple treatment.

#### 2.1.1. Decellularized Extracellular Matrix

Decellularized extracellular matrix (dECM) refers to biological materials formed by human or animal tissues or organs through decellularized methods [40]. Decellularized muscle matrix biomaterials were first reported in 1948; scientists have gradually deepened their research correlated with dECM in recent years [41,42,43,44,45]. Some dECM materials are FDA-approved, such as AlloDerm^®^ for skin repair [46]. Decellularized extracellular matrix refers to a kind of 3D culture framework that contains many extracellular macromolecules, including collagen, elastin, fibronectin, and matricellular proteins, creating a perfect regeneration microenvironment [47]. Furthermore, it retains some physicochemical properties and signal molecules obtained from the original tissues and provides a favorable carrier for recellularization [48]. Consequently, dECM has promising application prospects in damaged organ repair and tissue regeneration.

Decellularized pulp matrix from humans, swine, bovines, and rats have been experimented with using hypotonic or surfactant methods, exhibiting satisfactory decellularization efficiency [49,50,51,52,53]. Decellularized pulp retains major extracellular matrix components, such as type I collagen, type III collagen, laminin, and fibronectin [50]. Moreover, it contains some bioactive factors, including transforming growth factor β (TGF-β), dentin matrix protein 1 (DMP-1), and dentin sialoprotein (DSP) [51]. Furthermore, it retains the vascular structure and vascular endothelial growth factor (VEGF) and increases the relative mRNA expression level of angiogenesis markers during the treatment of recellularization [54]. Conditioned medium made from decellularized scaffolds can ameliorate the proliferation and migration ability of DPSCs, and the animal model of dental pulp regeneration demonstrates ideal results [51,54,55,56,57]. Decellularized pulp matrix also enhanced the odontogenic differentiation ability of DPSCs with high BMP4 expression with the help of recombinant adenovirus [58]. Considering the loss of extracellular matrix components during the acellular process, some researchers modified the decellularized dental pulp matrix materials with laminin, a kind of extracellular matrix protein, which exhibits more advantages in odontogenic differentiation [59]. Moreover, other soft tissue components of teeth, such as the tooth bud, indicate promising pulp regeneration potential after decellularized treatment [60,61,62].

Furthermore, the hydrogel derived from bone decellularized matrix showed a similar ability to promote odontogenic differentiation [63]. According to the related report, bovine bone tissue was treated to form 5 wt% dECM solution after decellularized treatment; then, the solution was mixed with DPSCs as part of the bio-ink to conduct 3D printing. As confirmed by experiments in vitro and in vivo, this 3D printing material has the characteristics of promoting bone/tooth differentiation [64]. Human amniotic tissue, rich in bioactive factors, forms a gel scaffold after decellularization that can sufficiently support the directional differentiation of DPSCs [65].

Apart from the description mentioned above, DPSCs-derived extracellular matrix has been reported. As revealed by relevant reports, such a matrix can enhance the mineralization level of dental pulp stem cells or gingival fibroblasts [66,67,68]. Regarding application, DPSCs can be seeded on another material, cultured for some time, and then treated with a decellularized method to form the decellularized composite material. For instance, DPSCs were cultured in a collagen/chitosan hydrogel for 2 weeks or grown on 3D-printed porous polylactic acid scaffolds for 7 days and then were decellularized [69,70]. These dECM-composites had favorable effects, indicating their potential application in pulp regeneration. DPSCs-derived extracellular matrix combined with colloidal microgels also enhanced the expression level of odontogenic genes in DPSCs [71]. Similarly, the extracellular matrix derived from other stem cells, such as stem cells from exfoliated deciduous teeth (SHEDs) or periodontal ligament stem cells (PDLSCs), also improved the mineralization ability of DPSCs [72].

The decellularized pulp matrix is obtained from the dental pulp tissue after decellularization, which retains most of the microstructures of the pulp tissue, carries some bioactive factors related to dental pulp regeneration, and largely preserves the original microenvironment of dental pulp cells, showing good potential for promoting pulp regeneration using bioactive scaffolds for the growth, proliferation, and differentiation of stem cells in clinical pulp regenerative therapy, despite the lack of clinical trial evidence to confirm it. Nonetheless, decellularized extracellular matrix materials have minor shortcomings, such as their complex steps, disinfection, batch-to-batch variation, and toxicity of residual decellularized liquids, which require further improvements to meet the needs of clinical practice [48].

#### 2.1.2. Treated Dentin Matrix

Treated dentin matrix (TDM) is made of dentin, the hard tissue that accounts for the most significant proportion of teeth after treatment with ethylenediaminetetraacetic acid (EDTA), which has a certain degree of demineralization, containing fully exposed dentin tubules and loose fiber bundles [73]. This material is abundant in proteins involved in dentin production, such as COL-1, DSP, TGF-β1, and DMP-1, suggesting that TDM can create a favorable microenvironment for dentin regeneration, confirmed by in vitro and in vivo experiments [74,75,76,77]. Further mechanistic studies revealed that the biological activity of TDM was achieved by regulating Wnt/β-catenin pathway [78]. SHEDs sheet-derived pellets with TDM are a promising strategy for pulp regeneration [79].

Treated dentin matrix paste (TDMP) could be used for pulp capping. TDMP could contribute to the odontogenic differentiation and create a more continuous dentin bridge in the dental pulp exposure model in miniature pigs [80]. Another improved TDM material, treated dentin matrix hydrogel, exhibited excellent results in direct capping [81,82]. TDM and stem cells have also been studied in periodontal tissue regeneration [83]. Apart from the previously introduced TDM (dentin treated with EDTA), lyophilized dentin had a similar effect [84].

Furthermore, a randomized controlled clinical trial involving 25 patients aged 7–12 years old with tooth avulsion caused by dental trauma also confirmed that decellularized dentin matrix combined with autologous DPSCs aggregates at the root of the tooth could effectively simulate the microenvironment of tooth development and support the continued root growth of young permanent teeth [85]. Similarly, TDM can also be used as a component of pulp-capping agent, which will play an influential role in direct or indirect pulp-capping clinical treatment.

In conclusion, the composition of TDM is similar to dentin, but its structure is looser than that of dentin. It contains and properly releases bioactive factors to create a microenvironment for pulp regeneration.

#### 2.1.3. Exosomes

Exosomes are extracellular vesicles generated by cells and contain nucleic acids, proteins, lipids, and metabolites, which play a vital role in intercellular communication and microenvironment creation [86,87]. Some studies have revealed that exosomes derived from dental stem cells favor promoting dental pulp regeneration [88,89]. Huang et al. [90] first isolated and identified DSPC-derived exosomes, and then confirmed that other DPSCs could endocytose these exosomes to activate the P38 MAPK pathway and have an odontogenic differentiation effect on DPSCs, which was also validated in animal models. Aside from that, exosomes derived from Hertwig’s epithelial root sheath cells, SCAPs, SHEDs, and Schwann cells could give an impetus to regenerating pulp dentin complex tissue [91,92,93,94,95].

As was reported in the relevant study, DPSCs-derived exosomes can enhance the angiogenic capacity of HUVECs [96]. Apoptotic vesicles from SHEDs stimulated vascular endothelial cells through the transcription factor EB-autophagy pathway and exerted similar effects [97]. LPS-pretreated DPSCs-derived exosomes make efforts to propel odontogenic differentiation of Schwann cells. Because exosomes are in small quantities and difficult to store, type I collagen membranes and fibrin gels could be loaded with exosomes to better simulate the microenvironment for in vivo experiments, which exhibits promising clinical applications [90,98].

Due to the complex production and the difficulty in storing and transporting exosomes, there are currently no reports on clinical trials in dental pulp regeneration [99]. Since exosomes participate in and contribute to a better regenerative microenvironment, they are waiting for follow-up research progress to meet the needs of clinical treatment and play an active role in cell homing and promoting stem cell proliferation in pulp regenerative treatment.

#### 2.1.4. Other Perspectives

Aside from the dECM, TDM, and exosomes mentioned above, other strategies utilize bioactive substances derived from natural ingredients to help create a microenvironment for regeneration.

Microvascular fragments isolated from adipose tissue ameliorated the vascularization and the dental pulp regeneration ability of DPSCs aggregates [100]. Regarding the construction of cell aggregates, Itoh et al. [101] scraped DPSCs from a culture plate. They filled the cell sheet in a gel model to obtain a rod-shaped 3D cell structure after culturing for 2 days, which then exhibited a satisfactory effect of dental pulp differentiation in related studies. In terms of clinical trials, 40 patients with pulp necrosis of young permanent teeth caused by tooth trauma were recruited. The researchers cultured DPSCs from autologous deciduous teeth to form aggregates and collected them into treated root canals. A two-year follow-up showed continuous development of tooth root and more blood flow signal through the ultrasound doppler test, which will be more widely used in clinical practice in the future [102]. There are problems in the preparation and application efficiency in forming cell aggregates, suggesting that a more extensive and convenient clinical application also needs better assistance from scaffold materials to achieve the curative effect of dental pulp regeneration.

As for platelets-derived bioactive substances, platelet lysates loaded with hyaluronic acid hydrogels or gelatin methacrylate (GelMA) microspheres also create a microenvironment to propel vascularized pulp regeneration [103,104]. Platelet-rich fibrin showed a more substantial regenerative potential of DPSCs, compared with platelet-rich plasma, mainly in LPS-induced inflammatory states, which has been confirmed by clinical trials of necrotic permanent teeth [105,106,107,108]. Studies have shown that placing autologous platelet-rich plasma or platelet-rich fibrin into the treated root canal could promote the continued development of the root apex of young permanent teeth, and it also promotes the restoration of pulp sensitivity to a certain extent when applied to mature permanent teeth with pulp necrosis [107,109,110].

Microvascular fragments, cell sheets, and blood component derivatives are abundant in bioactive factors, which can become part of the extracellular microenvironment to help regenerate dental pulp tissue.

### 2.2. Artificial Synthetic Material

Apart from the natural or naturally derived materials summarized above, synthetic materials can create a suitable microenvironment for tissue regeneration by assisting in 3D culture, simulating extracellular matrix, and loading bioactive factors.

#### 2.2.1. Agents for Creating a 3D Environment

As illustrated by relevant studies, the 3D culture of DPSCs can ameliorate the multidirectional differentiation potential and improve the mineralization level, helping to maintain the in vivo microenvironment of stem cells [111,112]. On that account, we primarily introduce three novel strategies to realize 3D culture.

##### Self-Assembled Peptide Hydrogel

Self-assembled peptide hydrogel is a bioactive peptide motif that forms an ordered structure on a micro-scale through molecular self-assembly. It can simulate the system of natural extracellular matrix to a certain extent, encapsulate bioactive factors with satisfactory biocompatibility and degradability, and show a promising application in tissue engineering [113,114]. Meanwhile, it is easy to achieve a 3D culture of cells because it is performed by self-assembling peptide motifs through non-covalent interactions [115].

Commercialized self-assembled peptide hydrogel, Puramatrix (RADA16-I), has been reported in dental pulp regeneration. For instance, DPSCs cultured in Puramatrix for 3 weeks could obtain odontogenic markers with high protein levels [116]. DPSCs alone or co-cultured with HUVECs in the same hydrogel exhibited vascularized pulp-like tissue in the subcutaneous model of nude mice for 4 weeks [117]. HIF-1α-stabilized SHEDs cultured in Puramatrix displayed ideal pulp regeneration effects in vivo and in vitro experiments [118]. Some researchers combined RAD with dentonin, a functional peptide motif of an extracellular phosphoglycoprotein, to form a functionalized self-assembling peptide hydrogel. As revealed by their research findings, it could give an impetus to the odontogenic differentiation of DPSCs [119]. Similarly, it has been reported that the self-assembled peptide hydrogels with RAD and VEGF mimetic epitopes have comparable results [120]. Using self-assembled hydrogels derived from the angiogenic peptide, SLan, to create an angiogenic niche has also been demonstrated to form dental pulp-like tissue in the canine orthotopic model [121]. Apart from that, the dentinogenic peptide is applied to self-assemble into hydrogels to support the growth of DPSCs, but it suffers from the problem of rapid degradation [122]. Similarly, self-assembled peptide hydrogels can cooperate with bioactive factors to create a pulp regeneration microenvironment. For instance, Mu et al. [123] constructed Puramatrix (RADA16-I) encapsulated with stem cell factor (SCF) to generate more blood vessel-like structures.

##### Microspheres

Microspheres are spherical polymeric networks with diameters ranging from about 100 to 400 microns, with sufficient oxygen diffusion capacity and a high surface area [124]. They can provide a 3D biomimetic environment and are commonly utilized in tissue regeneration and cell delivery [125]. As has been reported, different types of microspheres, such as nanofibrous spongy microspheres and gelatin methacrylate microspheres, were used to propel odontogenic differentiation of DPSCs and this achieved favorable results [126,127,128]. In line with related reports, unique microcapsules composed of co-encapsulation of DPSCs and HUVECs have been used for microstructural delivery in dental pulp regeneration [129,130].

Microspheres loaded with bioactive factors have also been reported, such as those containing the platelet lysates mentioned above [103]. The hierarchical nanofiber microsphere system loaded with VEGF or the gelatin methacrylate frozen gel microspheres loaded with simvastatin will be introduced in the following section of scaffold materials with bioactive factors [129,131,132].

##### 3D Printing

Apart from self-assembled hydrogel or microsphere systems, 3D printing technology is suitable for creating a comfortable 3D environment for cell culture. Park et al. [133] incorporated BMP-2 simulated peptides into gelatin methacrylate bio-ink and found it could give an impetus to the odontogenic differentiation of DPSCs after 3D printing. Decellularized dentin matrix or calcium silicate could be applied as part of bio-ink for 3D printing, exhibiting satisfactory effects of promoting odontogenic regeneration [134,135].

Altogether, the 3D culture of DPSCs is still in the developing stage. Furthermore, building a dental pulp organoid model with a complex microenvironment is one of the goals of tissue engineering in the future, as was reported on the organoid models of the intestine, lung, and other structures [136,137].

#### 2.2.2. Biomimetic Scaffolds

Because natural materials are limited by sources, costs, and mechanical properties, it is considered an excellent strategy to use bioactive materials that simulate extracellular matrix to provide an appropriate microenvironment. In line with research from Wang et al. [138], a poly-L-lactic acid nanofiber scaffold mimicking the structure of type I collagen can propel the odontogenic differentiation of DPSCs, showing a better effect if loaded with BMP-7. Similarly, Qu et al. [139] adopted nanostructured gelatin/bioactive glass to mimic collagen, which promoted the differentiation and biomineralization of DPSCs.

Moreover, a 3D tubular nanofiber matrix was created by adopting micropatterning technology to regulate the arrangement, migration, and differentiation of DPSCs, which helped obtain the dental pulp and dentin complex, which was analogous to the natural structure in the further in vivo experiments [140]. Based on the problem that DPSCs could not stably adhere to the root canal wall, some researchers, inspired by mussel adhesion, designed a dopamine-modified hyaluronic acid coating on the dentin surface to form an effective attachment bridge between DPSCs and dentin, which had a favorable performance on odontogenic differentiation ability [141]. Dopamine coating on the mineral trioxide aggregate (MTA) surface had a similar effect [142]. Consequently, biomimetic materials simulating extracellular matrix components or natural binding structures are widely used in tissue regeneration.

#### 2.2.3. Scaffolds Loaded with Bioactive Factors

As described in the first part, dental pulp restoration and regeneration is regulated by a series of bioactive factors. Therefore, bioactive factors could be introduced to provide a regeneration microenvironment to improve stem cells’ proliferation and directional differentiation ability during tissue engineering. Since bioactive factors are mainly ions, peptides, and proteins, the scaffold loaded with bioactive factors is expected to have stable concentrations. This better and more lasting effect makes this strategy more applicable in tissue engineering.

##### Bioactive Factors

The following list (Table 1) contains the common bioactive factors that regulate dental pulp regeneration.

##### Scaffolds Loaded with Bioactive Factors

Given the complicated clinical situation, the release of bioactive factors needs to consider such issues as activity, concentration, and effect, so it is essential to use scaffolds with specific biological effects to load bioactive factors to play the leading role. The standard methods of scaffolding loaded with bioactive factors include surface presentation, encapsulation, and layer-by-layer assembly [170,171]. Different loading strategies were selected based on the characteristics of materials and biological activity of factors, which showed satisfactory results.

➀ Surface presentation

The surface presentation can be achieved by physical adsorption or chemical binding to create the ideal microenvironment due to the unique morphology, functional groups, and charge on the material’s surface.

Based on the microporous structure and adsorption properties, silk protein was used for physical absorption of SDF-1α and slowly released the factor within 24 h after loading, exhibiting a good pulp regeneration effect [165]. In line with the relevant report, researchers loaded collagen with BMP-7, injected it into the root canal, and then added DPSCs to the root tip to simulate endogenous stem cells. They found that it could stimulate cell migration and propel regeneration of vascularized pulp tissue in subcutaneously nude mice models [172]. The 50 mm diameter polydioxanone fibers containing VEGF exhibited a good angiogenic effect [173]. Another example of the synergistic effect of this material with some bioactive factors is chitosan and its derivatives scaffolds; they were loaded with simvastatin, displaying the ability to improve the odontogenic differentiation and mineralization of DPSCs [174,175,176]. Regarding chemical binding, chitosan-loaded calcium ions through coordination bond is a good example; they continued to release calcium ions for 21 days, displaying potential applications in dental tissue engineering [177]. Like calcium ions, strontium could become part of nano-bioactive glass through the ionic bond and can be slowly released from the material, facilitating dentin formation in vivo studies [178].

The binding of cytokines to materials through chemical bonding has not been reported in dental tissue engineering. Although the number of bioactive factors adsorbed on the physical surface is limited, the experimental results are comparatively ideal, which adequately exhibits its potential clinical application.

➁ Encapsulation and layer-by-layer assembly

Encapsulation is an intelligent strategy that can protect the bioactive factors from premature degradation [170]. Some scholars have made relevant attempts. TGF-β and FGF2 were coated with degradable lactide and ethyl lactide polymers to form microspheres to continuously release the two factors and facilitate the proliferation and migration ability of DPSCs [144]. Porous chitosan microspheres containing TGF-β1 showed more restorative dentin in the pulp-capping model than the control one [179]. Li et al. [131] prepared a hierarchical nanofiber microsphere system encapsulated with VEGF, which simulated the structure of natural collagen fibers at the nanoscale. As demonstrated by their research findings, the VEGF in the system could be released slowly and uniformly within 4 weeks. Moreover, vascularized regenerated pulp tissue was successfully formed, accounting for over two-thirds of the entire root canal in the subcutaneous semi-in situ model of nude mice.

The materials mentioned above are primarily loaded with bioactive factors through the strategies of surface presentation or encapsulation according to their surface properties, thereby forming a favorable microenvironment for dental pulp regeneration. Meanwhile, endogenous bioactive factors can be released through special treatment to play their biological roles. For instance, endogenous TGF-β could be activated and released through low-intensity laser or injectable alkaline gel treatments, which is conducive to stimulating the differentiation of pulp dentin of DPSCs [16,180]. Apart from the traditional methods of loading bioactive factors, we expect that there will be new strategies for the design of scaffolds loaded with bioactive factors to simulate the structure of the extracellular microenvironment as well as the temporal and spatial distribution of bioactive substances based on the latest physical and chemical principles, to meet the needs of clinical practices better.

## 3. Novel Perspectives of Pulp Regeneration

Tissue regeneration rests on the interactions among scaffolds, bioactive factors, and stem cells. The above studies primarily concentrate on natural and naturally derived materials and artificial synthetic materials that simulate a microenvironment by creating a 3D culture environment or loading bioactive factors. The following content introduces different strategies for pulp regeneration from a novel perspective to better supplement the potential of the regeneration microenvironment.

### 3.1. Changing Physical Conditions

The biological activities of tissue cells are regulated by physical signals, such as matrix stiffness, magnetism, and light, which have been attempted as additional interventions for tissue engineering [181].

As revealed by research conducted by Qu et al. [182], DPSCs formed mineralized tissue on a 3D NF-gelatin scaffold with a high-stiff surface and pulp-like tissues on a low-stiff structure. They first designed a scaffold structure with a low-stiffness inner core and a high-stiffness surface and simulated the actual structure of teeth. Then, the subcutaneous experiments in nude mice revealed that the pulp dentin composite-like structure was successfully generated.

The external magnetic field can modulate the behavior of stem cells. As indicated in related studies, static magnetic fields can ameliorate the proliferation, migration, and odontogenic differentiation ability of DPSCs through the activation of YAP/TAZ and p38 MAPK signaling pathways [183,184]. Some researchers have adopted magnetic fields to control the transportation and relative location of cells [185]. For example, DPSCs and HUVECs were labeled with magnetic nanoparticles and the two cells were assembled layer by layer under magnetic control. The experimental results showed that the functional vascular network could be formed within 3 days, providing a fundamental basis for pulp regeneration [186]. The synergistic effect of graphene and pulsed electromagnetic fields on DPSCs can implement their neurogenic differentiation [187]. Future attempts can also be made to construct scaffolds containing endogenous magnetic fields to propel odontogenic differentiation of DPSCs, as described in a report where the magnetic layer drove the osteogenic differentiation of MC3T3 cells [188].

Likewise, photobiomodulation and optogenetics have been introduced to use DPSCs for regeneration. As the primary method of photobiomodulation, the low-level laser can enhance the odontogenic differentiation ability of DPSCs and SCAPs on scaffolds [189,190]. Arany et al. [16] found that low-level laser could activate endogenous TGF-β1 through reactive oxygen species. The in vitro and rat pulp-capping model experiments demonstrated that activated TGF-βs could improve the odontogenic differentiation ability of DPSCs. Regarding optogenetic regulation, Niyazi et al. [191] have transfected the light-sensitive protein, channelrhodopsins, which could activate the CaMKIIa (a kind of protein involved in nerve cell function) in DPSCs. After optical stimulation for 5 days and 90 min per day, it can propel the neural differentiation of DPSCs. Furthermore, the photothermal effect of gold nanoparticles is also adopted to elevate the mitochondrial metabolism and energy supply levels, enhancing the odontogenic differentiation of DPSCs [192].

Furthermore, the 3D microgravity culture system’s construction positively improves the proliferation and odontogenic differentiation of DPSCs [193,194]. The above physical conditions, such as matrix stiffness, magnetic field, and light stimulation, can ameliorate the survival-promoting potential of the microenvironment. The specific biological regulation needs further exploration.

### 3.2. Antibacterial Material

Root canal environments expected to realize pulp regeneration in clinical practice may still have residual bacterial toxin components, which raises an antibacterial requirement for pro-regenerative scaffolds, including hydrogels, nanomaterials, microspheres, etc. For instance, Afami et al. [112] developed functionalized hydrogels with cell adhesion motifs for the 3D culture of DPSCs, which had antibacterial activity against common bacteria in the root canal. As was precisely described in relevant studies [195,196], a low concentration of graphene oxide-copper composites can give an impetus to odontogenic differentiation of DPSCs while inhibiting the formation of biofilm from Streptococcus mutans. Meanwhile, pulp-capping materials with antibacterial and dentin regeneration properties are in considerable demand. Calcium–zinc–silicon-based micro-nano spheres have advantageous antibacterial effects. In particular, they can lessen the release of pro-inflammatory factors from M1 macrophages and give an impetus to DPSCs’ odontogenic differentiation [197]. The antibacterial property is easy to ignore in tissue regeneration materials; how to skillfully achieve the balance between antibacterial and stem cell regeneration remains further discussed.

### 3.3. Other Novel Viewpoints

Cerium oxide has a favorable function of scavenging active oxygen. In consideration of these properties, researchers made cerium oxide nanoparticles. They found that they could be internalized into DPSCs to play an antioxidant role and ameliorate cell viability, showing their potential applications in dental pulp regeneration [198].

Oxygen is the fundamental prerequisite for cell proliferation and differentiation during tissue regeneration. Oxygen diffuses into every corner of the tissue through dissimilar types of blood vessels in human tissue, supporting cell survival and physiological functions. Reviewing the existing literature, the current strategies to propel angiogenesis predominantly use scaffold materials with or without bioactive factors, as mentioned. Similarly, the co-culture of vascular endothelial cells (ECs) and dental pulp stem cells (DPSCs) can heighten their ability for odontogenic differentiation and angiogenesis [199]. In accordance with the relevant report, microtissue spheroids composed of HUVECs and DPSCs could successfully form vascular pulp-like tissue in vivo after transplantation in animal models [200]. In addition, making oxygen-containing hydrogels is also a preferable choice. Zou et al. [201] produced a gelatin methacrylate (GelMA) hydrogel containing calcium peroxide to release oxygen in situ to ensure the average growth of SCAP under a hypoxic environment.

Unlike the above small-scale clinical trials of natural or natural-derived materials such as PRF and TDM, artificial synthetic materials must undergo more stringent demonstrations and plans. Currently, there is no relevant clinical trial report, and we are waiting for further research.

## 4. Summary

In conclusion, we first reviewed the primary process and participating elements involved in dental pulp development and restoration, indicating that the microenvironment is vital for tissue regeneration. The microenvironment is composed of surrounding cells, insoluble extracellular matrix, and soluble bioactive factors, each of which can affect the state of stem cells to some extent and can exert a specific influence on the construction of a suitable regenerative microenvironment.

**Figure 2:**
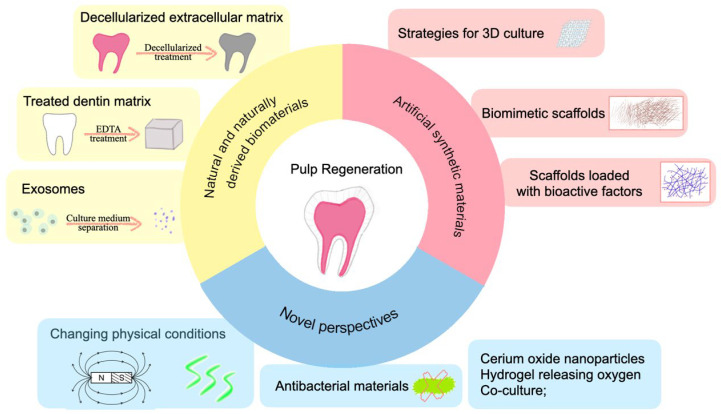
Summary of strategies for creating a microenvironment for dental pulp regeneration.

From the viewpoint of creating a microenvironment that propels tissue regeneration, the above studies summarized three topics containing natural and naturally derived materials, synthetic materials, and novel perspectives applied to materials (Figure 2). Natural and naturally derived materials consist of decellularized extracellular matrix, treated dentin matrix, exosomes, and platelet derivatives, which can simulate the microenvironment of tissue for the most part. This has a preferable effect on odontogenic differentiation. Nevertheless, some problems need to be solved. For example, it is not easy to mass produce and achieves homogenization between different batches. Artificial materials can overcome some of these problems quickly. Synthetic materials mainly consist of 3D culture scaffolds, biomimetic scaffolds, and bioactive factor-loading scaffolds, which primarily simulate the microenvironment by creating 3D culture conditions, imitating the morphology of the extracellular matrix or loading bioactive factors to help achieve pulp regeneration. As for novel perspectives, it contains materials or conditions that change the physical environment, have antibacterial effects, and elevate the oxygen content of tissue, which exhibits promising prospects in achieving pulp regeneration.

We put forth some strategies to assist in creating a microenvironment, hoping to inspire subsequent research on pulp regeneration. We look forward to more inspiring, practical, and clinically applicable strategies for pulp regeneration in the future.

## Figures and Tables

**Table 1:** Various bioactive factors involved in dental pulp regeneration.

Main Function	Bioactive Factors	Related Articles	Notes
Odontoblastic/Odontogenic differentiation	TGF-β1	[143,144,145]	Angiogenesis
	BMP2, BMP7	[146,147,148,149]	Extracellular Ca^2+^, Mg^2+^ can enhance their effects
	FGF	[18]	Angiogenesis
	IGF	[17,150]	Cell proliferation and migration
	EREG	[151]	/
	PDGF-BB	[152]	Migration capability
	Ca^2+^	[153,154]	
	Mg^2+^	[149]	/
	Sr^2+^	[155]	/
	miRNA	[156]	For example: miR-140-3p [157], miR-675 [158], etc.
Angiogenesis	VEGF	[159,160]	/
	HIF-1α	[161]	/
	miRNA	[93]	For example: miR-26a (SHED)
Neurogenesis	BDNF	[162]	/
	bFGF + NGF	[163]	/
	Nell-1	[164]	/
Chemotactic function	SDF-1α	[95,165,166]	Homing factors in dental pulp regeneration
	SCF	[166,167]	
	G-CSF	[168,169]	Ameliorate regeneration potential

## Data Availability

All data are included in this published review.
